# Optimal Implantation Site of Orthodontic Micro-Screws in the Mandibular Anterior Region Based on CBCT

**DOI:** 10.3389/fphys.2021.630859

**Published:** 2021-05-20

**Authors:** Yannan Wang, Quan Shi, Feng Wang

**Affiliations:** ^1^The Sixth Medical Center of PLA General Hospital, Beijing, China; ^2^Military Hospital, Qingdao, China; ^3^The General Hospital of People’s Liberation Army (301 Hospital), Beijing, China

**Keywords:** CBCT, mandibular anterior region, micro-screws, implantation site, post- implantation stability

## Abstract

**Background:** To determine the optimal implantation site of orthodontic micro-screws based on cone beam computed tomography (CBCT) analysis in the mandibular anterior tooth region, provide a theoretical basis for orthodontic implant placement and improve post-implantation stability.

**Methods:** Forty patients who underwent CBCT scanning were selected for this study. CBCT scanning was applied to measure the interradicular distance, buccolingual dimension, labial cortical bone thickness and lingual cortical bone thickness between mandibular anterior teeth at planes 2, 4, 6, and 8 mm below the alveolar ridge crest. The data were measured and collected to obtain a comprehensive evaluation of the specific site conditions of the alveolar bone.

**Results:** The interradicular distance, buccolingual dimension and labial cortical bone thickness between the mandibular anterior teeth were positively correlated with the distance below the alveolar ridge crest (below 8 mm). The interradicular distance, buccolingual dimension, labial cortical bone thickness, and lingual cortical bone thickness were all greater than those in other areas between the lateral incisor root and canine incisor root 4, 6, and 8 mm below the alveolar ridge crest.

**Conclusion:** The area between the lateral incisor root and the canine incisor root in planes 4, 6, and 8 mm from the alveolar ridge crest can be used as safe sites for implantation, while 8 mm below the alveolar ridge crest can be the optimal implantation site. An optimal implantation site can be 8 mm below the alveolar ridge crest between the lateral incisor root and the canine incisor root.

## Introduction

Most patients consider orthodontic treatment to not only correct the relative relationship of their teeth but also to improve their facial appearance with coordinating the functional relationship of their teeth, as anterior teeth play a large role in facial esthetics (Brend et al., 2020). For deep overbites of the maxillary anterior teeth that can lead to deep bites or excessively exposed gums, it is necessary to lower the upper anterior teeth. A steeper mandibular dentition Spee curve will cause a patient’s lower anterior segment to protrude, and a deeper mandibular Spee curve will cause a deeper overbite. Therefore, the mandibular anterior teeth need to be lowered to improve excessive covering, but for severely deep overbites, microscrews would be implanted into the alveolar bone of the anterior teeth of the upper and lower jaws to intrude the upper and lower anterior teeth, respectively, to obtain a more stable bite relationship. For patients with mild bites and no serious plane tilt, the corner of the mouth will be slightly tilted to one side to affects the appearance, and the bite needs to be lowered or raised to correct the patient’s vertical distance (Sugawara et al., 2008).

Orthodontic treatment should establish good occlusal contact and a stable occlusal relationship. Patients can receive many treatments, such as class II/III elastic traction, and of which anterior or posterior tooth pads can improve and stabilize the occlusal relationship (Saito et al., 2005). However, most orthodontic patients need effective means to lower the front teeth of the mandible to improve the occlusal relationship as soon as possible to avoid occlusal trauma. Proper treatments can solve multiple problems in the oral system, correct severe jaw deformities and address the unwillingness to undergo orthognathic surgery (Wang et al., 2017). A multidisciplinary approach can be applied to implement treatment to the extent possible to meet the major complaints of patients. The use of microimplant nail anchors can meaningfully correct of this type of deformity. Despite their small diameter and short length, they can still provide stable support for various tooth movements.

The effectiveness of orthodontics is dependent on the sustained and effective stability of the anchors. And factors that influence implant anchoring can be mainly classified into categories such as patient characteristics (age, gender, smoking habits, and dental hygiene condition), surgical operation (mechanical injury to bones from implant surgery, thermal injury), and micro-screw characteristics (diameter, length, implant angle, implant site bone density, and loading force duration; Kuroda and Tanaka, 2014; Sahoo et al., 2018; Sfondrini et al., 2018).

Operations to implant anchors are simple, but there is still a certain risk of surgical complications. In particular, due to the narrow interfurca distance, when the implant site is near the dental root or has a particular direction of deviation, the dental capsule and dental root may be injured, causing inflammation and even micro-screw loosening; therefore, it is very important to select the implant site accurately (Mohammed et al., 2018). To effectively use implant anchors, many researchers have made preoperative assessments of micro-screw implant sites by using cone beam computed tomography (CBCT). Their results have shown that based on CBCT, cortical bone thickness and sclerotin conditions could be effectively assessed, and the percentage of successfully implanted micro-screws could be effectively increased (Prasanpong et al., 2014; Qiu et al., 2016).

This research analyzes the safety margin and relative stable area of micro-screw implant sites for mandibular anterior teeth, based on the anatomic form measured by CBCT, to improve the initial stability of implant anchors and effectiveness of orthodontic treatments and to provide a reference for clinical operations.

## Materials and Methods

### Subjects

Forty patients who underwent CBCT scanning in our department from January 2017 to June 2018 were selected for this study, with a male to female ratio of 1:1 and an age range of 20 to 40 years. All subjects voluntarily signed the informed consent, and the study protocol was reviewed and approved by the Ethics Committee of the PLA General Hospital.

The inclusion criteria were as follows: (1) no orthodontic treatment history; (2) bilateral symmetry of the jaw bones; (3) good dental hygiene and no untreated or controlled periodontal disease or diseases of the oral mucosa; (4) overall health with no systemic disease that may influence bone metabolism; (5) X-ray film showing mandibular anterior teeth without obvious root resorption and with normal root morphology; and (6) presence of mandibular tooth 3–3.

The exclusion criteria were as follows: (1) anterior tooth crowding greater than 1.0 mm or severe rotation and declination; (2) root resorption or deformity; (3) periodontal disease in the mandibular anterior region or alveolar bone defect resulting from other factors; and (4) bone metabolism disease.

For the skeletal malocclusion in the research, there are 29 subjects of class I, and 11 subjects of class II, and no subjects of class III. Considering subjects for each class are few, so classification comparison research is not performed. However, for the future clinical research, the comparative research will be designed and analyzed.

### CBCT

**FIGURE 1:**
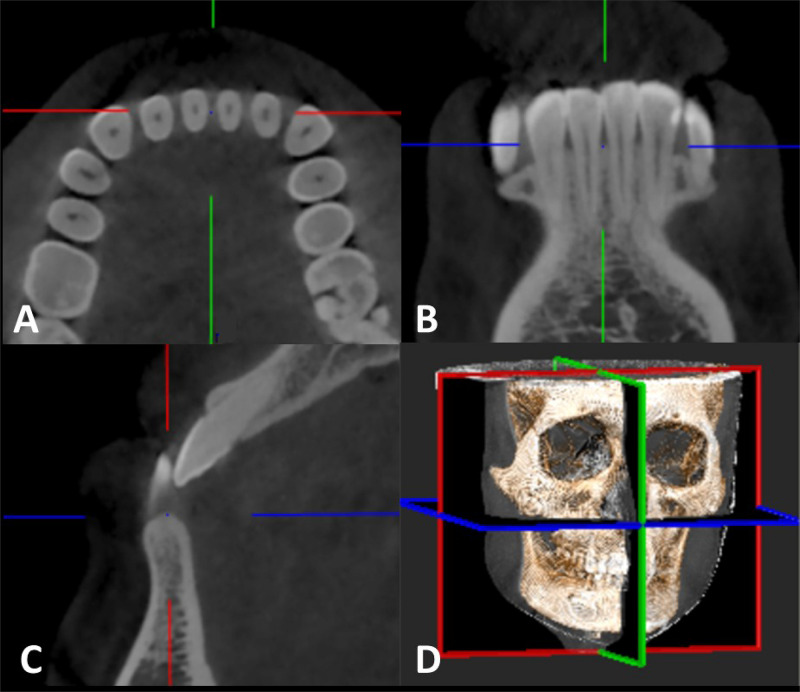
CBCT scanning and 3D reconstruction: **(A)** horizontal view; **(B)** coronal view; **(C)** sagittal view; and **(D)** three-dimensional reconstruction.

Scans of all patients were performed while the patients were held in a standing position. The head was adjusted to make the facial midline consistent with the median sagittal plane indication line and the orbitomeatal plane consistent with the horizontal plane indication lines. The lips were closed naturally, and the patient was asked to perform eupnea with no deglutition. All CBCT scans was performed with the same parameters, and three-dimensional reconstruction was performed using NNT Viewer v5.3 software with the scanning data, allowing observation of the coronal plane, sagittal plane, and horizontal plane in the mandibular anterior region. The steps were as follows: first, angles were adjusted to align the horizontal axis and the crest of the ridge between two dental roots; then, the sagittal plane was adjusted to align with the long axis of the teeth to measure the buccolingual alveolar bone thickness and cortical bone thickness; finally, the coronal plane was adjusted to align with the crest of the ridge to measure the interradicular distances between dental roots. The scanned data were saved under the DICOM 3.0 standard and were analyzed and three-dimensionally reconstructed by a CBCT image analyzer, as shown in Figure 1.

### Measurement Method

**FIGURE 2:**
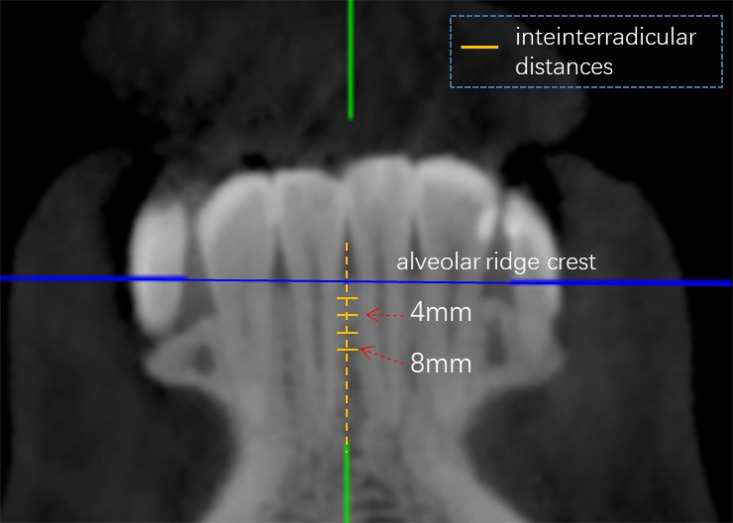
CBCT measurement: interradicular distance in coronal view.

**FIGURE 3:**
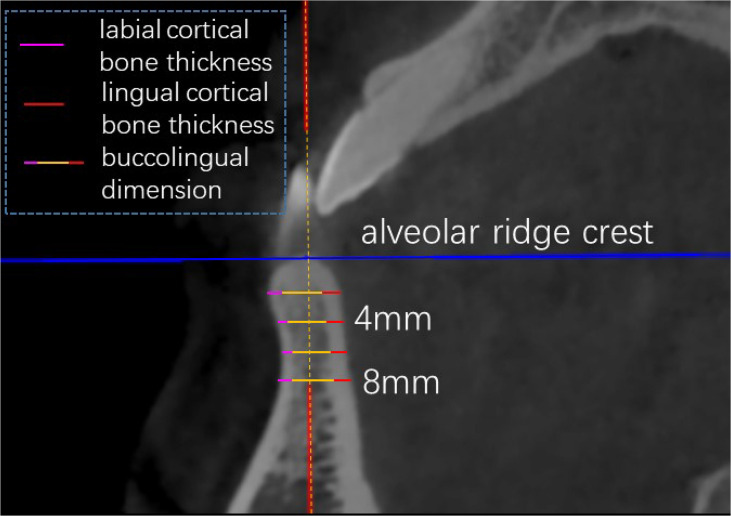
CBCT measurement: buccolingual dimension, labial cortical bone thickness and lingual cortical bone thickness in sagittal view.

Cone beam computed tomography scanning was applied to measure the interradicular distances between neighboring roots, buccolingual dimensions, labial cortical bone thickness, and lingual cortical bone thickness between maxillary anterior teeth at planes 2, 4, 6, and 8 mm below the alveolar ridge crest of all patients, as shown in Figures 2, 3. The measurement was repeated after 2 weeks, and the average was taken between the two measurements.

### Statistical Analysis

SPSS 19.0 software was used for the statistical analysis. The measurement data (represented as the mean ± SD) were analyzed with repeated-measures ANOVA using position and distance as independent variables, and two-sample *t*-test was used for gender; pairwise comparison LSD *t*-tests were used for *post hoc* analysis for position, and pairwise difference *t*-test was used for *post hoc* analysis for distance. *P* < 0.05 indicates statistically significant differences.

## Results

### Gender Difference

The scanning data between male and female was analyzed with two-sample *t*-test, and the result listed in Table 1 shows no statistically significant differences for interradicular distance, buccolingual dimension, labial cortical bone thickness, and lingual cortical bone thickness. So the measurement data of male and female are combined for the below analysis.

**TABLE 1:** Comparison result of male and female scanning data.

Alveolar bone	*P*	*t*
Interradicular distance	0.4346	–0.8200
Buccolingual dimension	0.1241	–1.6778
Labial cortical bone thickness	0.5520	0.1214
Lingual cortical bone thickness	0.6730	–0.2500

### Interradicular Distances Between Dental Roots

The scanning data of alveolar bone interradicular distances in the mandibular anterior tooth region are listed in Table 2. The overall comparisons (repeated-measures ANOVA) show that the effects of position, distance, and their interaction are all significant (*P* < 0.05). Detailed pairwise comparison results coupled with the data show that the interradicular distance between the mandibular anterior teeth is positively correlated with the distance below the alveolar ridge crest. Between the lateral incisor and canine, at 8 mm below the alveolar ridge crest, interradicular distances reached a maximum value of 2.77 mm. Between the central incisor and lateral incisor, at 2 mm below the alveolar ridge crest, interradicular distances reached a minimum value of 1.38 mm.

**TABLE 2:** CBCT results of the mesiodistal dimension in the mandibular anterior tooth region (mm, $\bar{x}\pm \text{s}$).

Position	1–1	1–2	2–3
2 mm	1.47 ± 0.51	1.38 ± 0.30	1.95 ± 0.51ab
4 mm	1.55 ± 0.63	1.49 ± 0.71	2.17 ± 0.65abc
6 mm	1.68 ± 0.70c	1.47 ± 0.49c	2.45 ± 0.70abc
8 mm	1.98 ± 0.90c	1.60 ± 0.57ac	2.77 ± 0.86abc

*a *P* < 0.05, vs 1–1; b *P* < 0.05, vs 1–2; and c *P* < 0.05, vs 2 mm. HF, Huynh-Feldt. Central incisors: 1–1, central incisor and lateral incisor: 1–2, and lateral incisor and canine teeth: 2–3.*

### Buccolingual Dimension Between Dental Roots

The results listed in Table 3 for the analysis of the buccolingual dimension show that the effects of position, distance, and their interaction are all significant (*P* < 0.05). A detailed pairwise comparison coupled with the data shows that the buccolingual dimension between the mandibular anterior teeth is positively correlated with the distance below the alveolar ridge crest. Between the lateral incisor and canine tooth, at 4 mm below the alveolar ridge crest, the buccolingual dimension reached a maximum value of 7.04 mm. Between the two central incisor teeth, at 2 mm below the alveolar ridge crest, the buccolingual dimension reached a minimum value of 4.99 mm.

**TABLE 3:** CBCT results of the buccolingual dimension in the mandibular anterior tooth region (mm, $\bar{x}\pm \text{s}$).

Position	1–1	1–2	2–3
2 mm	4.99 ± 0.75	5.88 ± 0.81a	6.64 ± 0.80ab
4 mm	5.29 ± 0.94c	6.17 ± 0.94ac	7.04 ± 0.92abc
6 mm	5.40 ± 0.80c	5.91 ± 0.82a	6.76 ± 0.98ab
8 mm	5.76 ± 0.90c	5.95 ± 0.87	6.82 ± 1.12ab

*a *P* < 0.05, vs 1–1; b *P* < 0.05, vs 1–2; and c *P* < 0.05, vs 2 mm.*

### Labial Cortical Bone Thickness Between Dental Roots

The results listed in Table 4 for the analysis of labial cortical bone thickness show that the effects of both position and distance are significant (*P* < 0.05), while their interaction is not significant. The labial cortical bone thickness between the mandibular anterior teeth is positively correlated with the distance below the alveolar ridge crest. Between the lateral incisor and canine, at 8 mm below the alveolar ridge crest, the labial cortical bone thickness reached a maximum value of 1.52 mm. Between the two central incisor teeth, at 2 mm below the alveolar ridge crest, the labial cortical bone thickness reached a minimum value of 0.97 mm.

**TABLE 4:** CBCT results of labial cortical bone thickness in the mandibular anterior tooth region (mm, $\bar{x}\pm \text{s}$).

Position	1–1	1–2	2–3
2 mm	0.97 ± 0.25	0.99 ± 0.26	1.23 ± 0.27ab
4 mm	0.97 ± 0.26	0.97 ± 0.22	1.25 ± 0.30ab
6 mm	0.98 ± 0.29	1.05 ± 0.23	1.33 ± 0.27abc
8 mm	1.18 ± 0.35c	1.21 ± 0.30c	1.52 ± 0.30abc

*a *P* < 0.05, vs 1–1; b *P* < 0.05, vs 1–2; and c *P* < 0.05, vs 2 mm.*

### Lingual Cortical Bone Thickness Between Dental Roots

Only the effect of position was significant (*P* < 0.05) in the analysis of lingual cortical bone thickness, as shown in Table 5. The lingual cortical bone thickness between the mandibular anterior teeth is positively correlated with the distance below the alveolar ridge crest. The lingual cortical bone thickness between the lateral incisor and canine is larger than the bone thicknesses between all other teeth at the same plane of distance below the alveolar ridge crest. At 8 mm below the alveolar ridge crest, the thickness reached a maximum value of 2.30 mm. Between the central incisor and lateral incisor, at 2 mm below the alveolar ridge crest, the lingual cortical bone thickness reached a minimum value of 1.27 mm.

**TABLE 5:** CBCT results of lingual cortical bone thickness in the mandibular anterior tooth region (mm, $\bar{x}\pm \text{s}$).

Position	1–1	1–2	2–3
2 mm	1.40 ± 0.29	1.27 ± 0.23	1.74 ± 0.41ab
4 mm	1.51 ± 0.32c	1.36 ± 0.27	1.98 ± 0.45abc
6 mm	1.64 ± 0.28c	1.50 ± 0.24c	2.14 ± 0.43abc
8 mm	1.78 ± 0.34c	1.78 ± 0.31c	2.30 ± 0.43abc

*a *P* < 0.05, vs 1–1; b *P* < 0.05, vs 1–2; and c *P* < 0.05, vs 2 mm.*

## Discussion

Some articles point out that implanting micro-screw into the mandibular premolar area is a safe and effective method with good treatment effect. While the mandible anterior area has fewer important anatomical structures and is not easily damaged. There are few researches on the safety of micro-screw into the anterior mandibular region (Ishihara et al., 2013). Common oral deformities, such as deep overbites and bimaxillary protrusion, directly influence patients’ facial morphologies. Deep overbites resulting from mandibular anterior alveolar bone overdevelopment are normally corrected by lowering the anterior teeth. For patients with bimaxillary protrusion, adduction and reduction of the anterior teeth can effectively improve the facial shape. Strong anchoring is fundamental to ensure anterior teeth adduction. The use of effective anchors has become an important method for correcting bimaxillary protrusions after the extraction gap is closed (Mimura, 2008; Teng et al., 2016).

Orthodontic micro-screws have become popular research tools for scholars at home and abroad because of their small size, high comfort, simple implantation method, shortened treatment time, and ability to meet the requirements of absolute anchoring. It is difficult to rely on imaging methods to evaluate the safety of sites for micro-screw implanting. Conventional X-ray film measurement can result in severe distortions and overlapping dental images, often resulting in failure to obtain cross-sections between roots, and cannot be used to measure bone thickness. In contrast, CBCT yields high-resolution images with a clear field of view and has high scanning efficiency while requiring a low radiation dose, and results in few scanning artifacts. CBCT can be used for effective evaluation of the alveolar bone condition at the site of the implanted micro-screw and the positional relationship with adjacent anatomical structures, making it possible to avoid damage to important anatomical structures and provide a reference for clinical applications (Poon et al., 2015). In addition, CBCT scans can yield spatial information about the maxillofacial region, allowing 3-d images to be reconstructed by a computer to perform measurements in the maxillofacial region (Min et al., 2012; Diniz-Freitas et al., 2015). Studies have shown that the stability of the micro-screw can be influenced by the choice of the implantation site, the bone condition at the implant site and the distance from the adjacent teeth. The application of CBCT will increase the accuracy of implantations and reduce the failure rate (Yaqi et al., 2016; Tepedino et al., 2018).

Micro-screw implants are generally suitable for adult patients. Because the bone tissue of juvenile patients is in the active phase, the reconstruction of the bone tissue after loading is unstable, and bone resorption is enhanced. The bone tissue of adults is relatively stable, and bone reconstruction is gentle after implantation of micro-screws, thus improving the stability of the planting anchors. Conventional implantation sites for micro-screws are generally selected between two roots (Chang and Tseng, 2014). For the anterior region, micro-screws are generally used to solve problems such as mandibular protrusion and deep lamination. Therefore, the conventional implantation site is between the two central incisors, between the central incisors and the lateral incisors, or between the lateral incisors and the canines (Ludwig et al., 2011). According to the data analysis of this experiment, the interradicular distance, buccolingual dimension, and buccal lingual bone thickness between maxillary anterior teeth were positively correlated with distances below the alveolar ridge crest below 8 mm. Generally, between 2 and 3, the interradicular distance is the widest (approximately 2.77 mm) at 8 mm below the alveolar ridge crest. However, the choice of micro-implant diameter is often limited in the clinic. If the diameter is too large, cracks will occur in the cortical bone, which will affect stability. Therefore, when micro-screws are implanted in the mandible, it is recommended for implants generally selected in the clinic to have a diameter between 1.0 and 2 mm. Between 2 and 3, at both 4 and 8 mm below the alveolar ridge crest, the buccolingual dimensions are wide, with dimensions of approximately 7.04 and 6.82 mm, respectively. Therefore, the length of micro-screws should not exceed 6 mm in clinical practice (Sarul et al., 2014); otherwise, due to its thickness and interradicular distance, the alveolar bone may be damaged by the micro-screw. Furthermore, if the micro-screws are too close to the root, they will cause loosening and shedding, resulting in failure of the implantation surgery and damage to other important anatomical structures due to incorrect positioning. Therefore, the micro-screw should be positioned in the central area between the two roots to ensure the continuity and stability of the anchor (Cornelis et al., 2007; Shigeeda, 2014; Xiang et al., 2018). In clinical practice, it is necessary to consider the proper safety range of both the alveolar bone and the soft tissue. The closer the implant anchor is to the edge of the lip mucosa, the more friction is encountered, and the more difficult it is to maintain oral hygiene, causing the planting anchor to fall off due to inflammation (Aras and Tuncer, 2016; Michele et al., 2017). At 2 mm below the alveolar ridge crest, the interradicular distance and buccolingual dimension between the two central incisors are 2.700 and 4.99 mm, respectively, and those between the central incisors and lateral incisors are 1.38 and 5.88 mm, respectively. Avoiding this area during clinical operation is recommended.

The mechanism of implant anchorage correction for a deep overbite is mainly achieved by the depression of the lower anterior teeth. Studies have confirmed that desired results can be achieved in patients with deep overbite implants with anchors implanted between the two roots of the mandible, using a 100 g force for 3 to 6 months. Therefore, when implants are inserted into the mandible, the results are influenced by important factors such as the values of depression, depression time, and traction direction.

The implantation angle of micro-screws can significantly affect the stress of the cortical bone. As the implantation angle increases, the thickness of the cortical bone gradually decreases, the torque of the micro-screws increases gradually, and the shedding rate increases (Rodriguez et al., 2014). Some studies have shown that the distance between adjacent roots is large, requiring an implantation angle of 90°; when the spacing between adjacent roots is small, the initial stability of the micro-screws can be significantly improved when the implantation angle is 60°–70° (Yong-Qing and Yi-Ming, 2011). In addition, the thickness of the cortical bone is positively correlated with the mechanical fitting force at the implanted screw-bone joint surface. In principle, the success rate of surgeries is significantly improved when the thickness of the cortical bone at the implant site is greater than 1 mm (Zheng et al., 2016). Therefore, the patient’s cortical bone should be measured before surgery. If the patient’s cortical bone is thin, the appropriate implantation angle and implantation direction should be selected to improve the stability of the micro-screw. The experimental data from this study showed that between 2 and 3, the labial and lingual bone thicknesses were thickest 8 mm from the alveolar crest, and the stability was the highest; between 1 and 1, the buccolingual di mension was short, but the interradicular distance was still acceptable; and between 1 and 2, the interradicular distance was acceptable, but the buccolingual dimension was short. Caution should be taken if implants are required in this area, and it is recommended to design a suitable implantation method to improve the stability of the anchor.

Due to anatomical structural limitations, the anterior region of the mandible is rich in blood vessels for the lingual nerve tissue, and the bone in that region is weak. It is difficult to implant an anchor in this location, and an implant can easily fall off. Additionally, the buccal cortex of the mandible is thick, and the depression is more difficult to manage than is the depression of the maxillary anterior teeth. After depression, the mandibular anterior teeth are more likely to undergo root resorption. Changes in alveolar bone 8 mm from the alveolar crest were noted in this study, and the effects of the labial mucosa and ligaments on the experimental data were not considered. Because the implant supports the lower anterior teeth, the angle of traction may have affected the results of the study, causing the anterior labia to tilt. In addition, the low-pressure value and the depression time are also important factors affecting the results. In future studies, the effects of the condition of the patient’s labial soft tissue and the direction of the traction force on anchor placement will be analyzed.

## Conclusion

In summary, CBCT-based measurements of the mandibular anterior region showed that within the allowable range of the soft tissue, the interradicular distance, buccolingual dimension, and labial and lingual bone thicknesses were all wide at a plane 8 mm below the alveolar ridge crest. In this area, micro-screws with diameters of 1–2 mm and lengths of no more than 6 mm can be selected in the clinical setting. The thickness of the labial bone is the least between 1 and 1, and a suitable implant method should be employed when implanting micro-screws to improve their stability. Between 1 and 2, at a plane 2 mm below the alveolar ridge crest, the interradicular distance is the shortest, approximately 1.38 mm. If a micro-screw is to be implanted here, care should be taken to select the material with the appropriate specifications.

## Data Availability Statement

The raw data supporting the conclusions of this article will be made available by the authors, without undue reservation.

## Ethics Statement

The studies involving human participants were reviewed and approved by the Ethics Committee of the PLA General Hospital. The patients/participants provided their written informed consent to participate in this study. All subjects voluntarily signed the informed consent.

## Author Contributions

YW and QS designed the study in consultation with FW. All authors assisted with sample collection. YW and QS conducted data acquisition and analysis. YW drafted the article and revised with other authors. All authors have read and approved the manuscript.

## Conflict of Interest

The authors declare that the research was conducted in the absence of any commercial or financial relationships that could be construed as a potential conflict of interest.
